# Nanosecond Laser Etching of Aluminum-Plated Composite Materials Applied to Frequency Selective Surfaces

**DOI:** 10.3390/ma13122808

**Published:** 2020-06-22

**Authors:** Jian Cheng, Shufeng Jing, Deyuan Lou, Qibiao Yang, Qing Tao, Zhong Zheng, Lie Chen, Xuefeng Yang, Dun Liu

**Affiliations:** 1 School of Mechanical Engineering, Hubei University of Technology, Wuhan 430068, China; chengjian@hbut.edu.cn (J.C.); longlaser@126.com (S.J.); loudeyuan@hbut.edu.cn (D.L.); yangqibiao@hbut.edu.cn (Q.Y.); taoqing107@aliyun.com (Q.T.); zhengzh215@163.com (Z.Z.); cangn1983@163.com (L.C.); 2 School of Mechanical Engineering, Jinan University, Jinan 250022, China; me_yangxf@ujn.edu.cn

**Keywords:** nanosecond laser, composite material, incident angle, pulse overlap, frequency selective surface

## Abstract

High-quality frequency selective surfaces (FSSs) are important for electromagnetic signal absorption/filtration. Usually, they are made from wave-transparent composite materials covered with a thin metal layer. Current machining methods show some disadvantages when performing fabrication on the structure. Based on its flexibility and uncontactable processing characteristics, nanosecond laser etching of aluminum-plated composite materials applied to FSSs was investigated. To observe the influence of the laser light incident angle, etching of a series of square areas with different incident angles was performed. Thereafter, an image processing method, named the image gray variance (IGV), was employed to perform etching quality evaluation analysis. The observed microscopic pictures of experimental samples were consistent with those of the IGV evaluation. The potential reasons that might affect the etching quality were analyzed. Following all the efforts above, an incident angle range of ±15° was recommended, and the best etching result was obtained at the incident angle of 10°. To observe the influence of the laser pulse overlap and focal spot size on the etched area border uniformity and on the potential damage to the base materials, a theoretical equation was given, and then its prediction of area border edge burrs fluctuation was compared with the experiments. Furthermore, SEM pictures of etched samples were examined. Based on the study, a processing window of the laser pulse overlap and focal spot size was recommended. To conclude, optimal etching results of the FSS materials could be guaranteed by using the right laser operating parameters with the nanosecond laser.

## 1. Introduction

Frequency selective surfaces (FSSs) are widely used in aeronautical, astronautical and warship facilities due to their excellent electromagnetic signal absorption/filtration performance [1,2,3,4,5]. For instance, Shang demonstrated FSS validation on radar cross-section reduction [6], while Li showed that FSSs could be used as meta-surfaces for cloaking and stealth purposes [7]. Recently, research on possible application in the THz spectrum has been reported, indicating its potential remote-sensing function [8]. Additionally, Zhang’s study enhanced this approach by designing a multi-layer FSS and controlling the chemical potential of a graphene layer [9]. Furthermore, they could also be applied to forthcoming 5G communication to improve signal transmission performance [10]. Usually, FSSs are made from wave-transparent composite materials covered with a thin metal layer. To achieve the electromagnetic signal absorption/filtration function, the metal layer should be machined into high-accuracy periodic patches without damaging the based wave-transparent materials. Wave-transparent materials include glass fiber composites, silica fiber composites, foam, polyimide (PI), etc. [4,11,12,13]. Additionally, gold, copper and aluminum are usually selected as candidates for the top thin metal layer [8,14,15].

Many different types of machining methods, ranging from computer numerical controlled (CNC) milling to chemical etching, and even continuous lasers and pulsed lasers, have been reported to perform FSS parts fabrication [16,17,18]. A 5-axis robot CNC milling machine has been employed by Zhu et al. to successfully process a 38-μm copper layer on FSSs [16]. Kim adopted an e-beam evaporator to form the copper-layer pattern on a glass/epoxy prepreg and thus fabricated a new kind of FSS [19]. FSSs could also be printed on dielectric layers using a resistive ink made of a suspension of carbon nanotubes [20]. An Nd:YAG laser was used by Zeng et al. to etch a ceramic substrate [21], and ultrafast femtosecond laser micromachining of two concentric hexagon-shaped metal slots on a Teflon substrate has also been investigated [8]. Femtosecond laser etching is a promising technique; however, its complicated optical delivery system and high cost will block its quick and wide application in FSS fabrication at least for the next 1–2 years.

With its high flexibility of processing, high energy density and strong adaptability of materials, nanosecond fiber laser usage and study have become more and more popular. For instance, Hua used a 100-watt nanosecond laser to do aluminum-oxide-layer cleaning [22]. Zhang et al. simulated the temperature distribution of metal thin films on a PI substrate under nanosecond laser irradiation, and their results were instructive and helpful to understand the actual laser etching process [23]. A method to fabricate and characterize terahertz frequency selective surface filters from a low-cost silver adhesive tape with a 20-nanosecond laser has been reported in [24]. However, nanosecond laser processing showed some thermal effects and accuracy shortages due to its pulse duration and improper processing procedures.

Taking the disadvantages above into account, the main aim of this work is to study the limitations of three key operating parameters that may determine nanosecond laser etching quality and accuracy, i.e., to clarify the influence of the laser incident angle, focal spot size and pulse overlap. The samples used were silica reinforced resin matrix composites with a 20-μm-thick aluminum film on top, provided by an aviation company in Jinan, China. An IPG nanosecond laser with a 100-ns pulse duration was adopted to perform the etching test. Thereafter, the influence of the laser light incident angle on etching quality and the influence of the pulse overlap and focal spot size on etching accuracy and uniformity were studied experimentally and theoretically. Since FSS materials have similar structures, the possible findings in this study could hopefully be applied for different FSS material compositions.

## 2. Experimental Procedure

### 2.1. Materials

**Figure 1:**
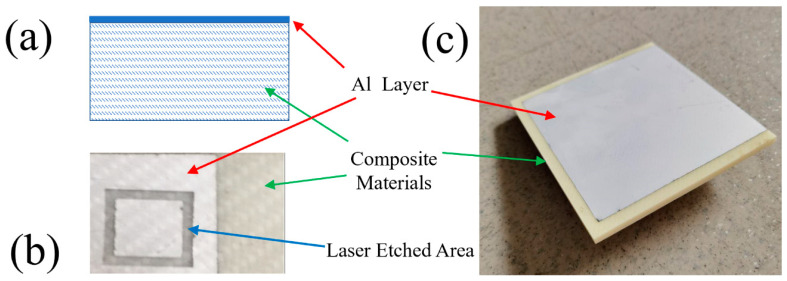
A cross-section schematic of the studied composite sheet (**a**), a top view of the sample (**b**) and the raw sample sheet (**c**). The thicknesses for the Al layer and the composite materials were 20 μm and 4.2 mm, respectively.

Commercially available aluminum-plated silica-reinforced resin matrix composite sheet (see Figure 1), which is a kind of raw material for FSS parts, was given by an aviation industry company in Shandong (Jinan, China). The sheet was then cut into 100 × 100 mm pieces. The aluminum layer was about 20 μm thick, and the silica reinforced resin matrix composite was about 4.2 mm thick. Nanosecond laser etching tests were then executed on the cut pieces.

### 2.2. Experimental

**Figure 2:**
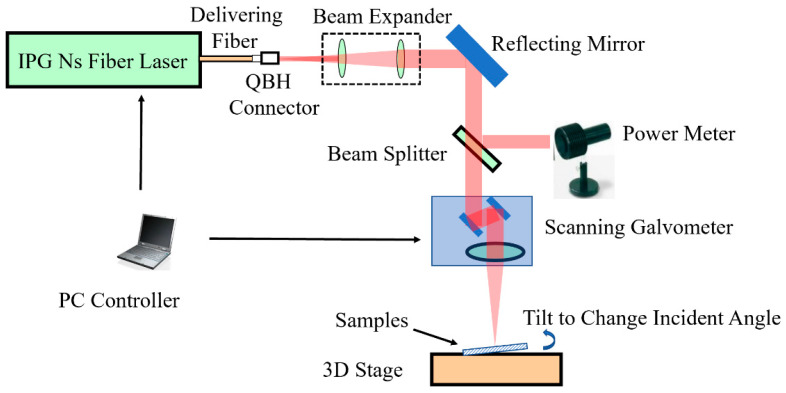
A schematic of the experimental setup. With a PC controller, the output laser beam and the action of the scanning galvanometer could be well synchronized. By raising one side of the samples and then measuring with a level meter, the incident angle could be tuned.

Laser etching experiments were performed by using a nanosecond fiber laser (IPG YLP-100, IPG Laser GmbH, Burbach, Germany). Figure 2 is the schematic diagram of the laser etching system. Briefly, it consisted of a laser source, an optical delivering system, a scanning galvanometer and an *x*–*y*–*z* three-dimensional stage. The laser source was an IPG nanosecond laser with a wavelength of 1064 nm, a pulse width of 100 ns (Full Width at Half Maximum), a maximum output power of 100 watts and an *M*^2^ ≤ 1.3. To observe the influence of the laser light incident angle on etching quality, samples on the stage were tilted from 0° to 35° to etch 1 × 1 mm squared areas, assuring the maximum height difference ≤ depth of focus. The aluminum-layer ablation threshold was tested to be 3.15 J/cm^2^ by using a single-pulse drilling method, which has been mentioned in [25]. The single-pulse drilling study on Al with a nanosecond laser was done by Zhang et al. recently. Their study showed that melting happened, and the heat-affected zone (HAZ) increased with the pulse energy density in a logarithmic pattern when doing laser irradiation [26]. In reference to that paper, the laser fluence adopted was only several times that of the threshold energy, so as to hinder the HAZ. All parameters used during the laser etching are shown in Table 1, with the number of overscans being 1. To observe the influence of the focal spot size and pulse overlap on the etching accuracy and etched area border uniformity, two different focal spot sizes of 32 and 50 μm were tested and compared, consequently. The laser parameters used during the laser etching are shown in Table 2. After laser processing, the surface profiles of the as-prepared and etched composite materials were observed via a Nikon 3100 optical microscope (Nikon Instruments Inc, Tokyo, Japan) and a Zeiss Gemini 300 SEM system (Carl Zeiss Microscopy GmbH, Jena, Germany).

## 3. Results and Discussion

### 3.1. The Influence of Laser Incident Angle on Etching Quality and Accuracy

In the case of flat-surface laser etching, it is not very difficult to find fixed laser processing parameters for high-accuracy results. However, FSSs are not regular flat surfaces. Indeed, most FSSs are three-dimensional freeform surfaces that hugely increase the difficulty of uniform etching. With this condition considered, tolerant windows for main laser processing parameters are required. One key factor that may seriously affect the etching results is the laser light incident angle. Therefore, experimental results and some discussions of the effect of the laser light incident angle were provided. In this case, the maximum height difference could be calculated to be 0.57 mm via 1 mm × sin35° = 0.57 mm. The depth of focus (*DOF*) for the optical system was ±1.4 mm, according to Equation (1) [27].

(1) $$DOF=\pm 2.56{f\;}^{2}M^{2}\lambda$$

where ${f\;}^{2}$ is the ratio of focal length to the expanded laser beam diameter, *M*^2^ is the beam quality value and *λ* is the laser wavelength. Therefore, the laser fluence could be regarded as uniform in vertical direction in this experiment.

**Figure 3:**
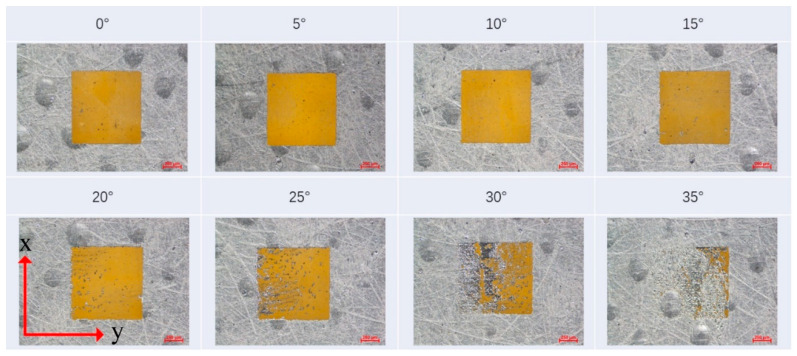
Etching evolution pictures captured with an optical microscope for the increased laser incident angle from 0 to 35°. The etched area was a series of 1 × 1 mm squares.

Figure 3 shows the etching evolution diagram with an increased laser incident angle under previously mentioned parameters in Table 1. It can be seen that laser etching uniformity improved a little from 0 to 5 and 10°, and then reversed gradually to 20°. With the incident angle further increasing, non-etching areas appeared from the left sides and quickly grew up to the right sides. Finally, the etching quality deteriorated to an unacceptable level.

In order to further characterize the surface etching quality, an image processing method, named the image gray variance (IGV) [28], was adopted for the evaluation. With IGV, selected sample surfaces were captured by using a microscope under the same light angle and intensity. Image pixel gray levels were collected, and then gray variances were calculated with Equation (2). Usually, the smaller the IGV value, the better the uniformity of the image, which means a better laser etching quality was achieved in this case.

(2) $$S=\frac{1}{MN}\sum_{i=1}^{M}\sum_{j=1}^{N}{\left[f\left(i,j\right)-\bar{f}\right]}^{2}$$

**Figure 4:**
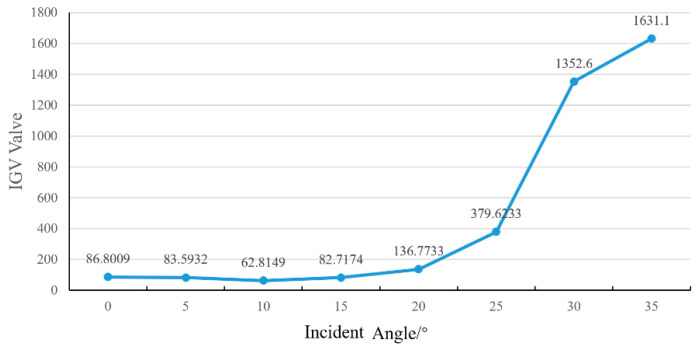
The image gray variance values changing with the incremental incident angle.

In the equation, *S* is the IGV value of the captured area, *f(i,j)* is the gray level of a pixel and $\bar{f}$ is the averaged gray level of the area, and *M* and *N* are the sampling numbers in *x* and *y* directions, respectively. The analysis with the IGV method is given in Figure 4. Generally, the trend line of IGV coincides with the observed results in Figure 3 very well. A minimum IGV value of 62.81 was achieved at the laser light incident angle of 10°; comparably, a maximum IGV value of 1631.1 was achieved at the laser light incident angle of 35°. There were some slightly differences between the angles of 0 to 15°. After 15°, the slopes of the line graph rose very fast, which meant unstable etching conditions. Hence, for a 1 × 1 mm area, a 30° (from −15 to +15°) processing window for laser light incident angle could be clearly inferred.

**Figure 5:**
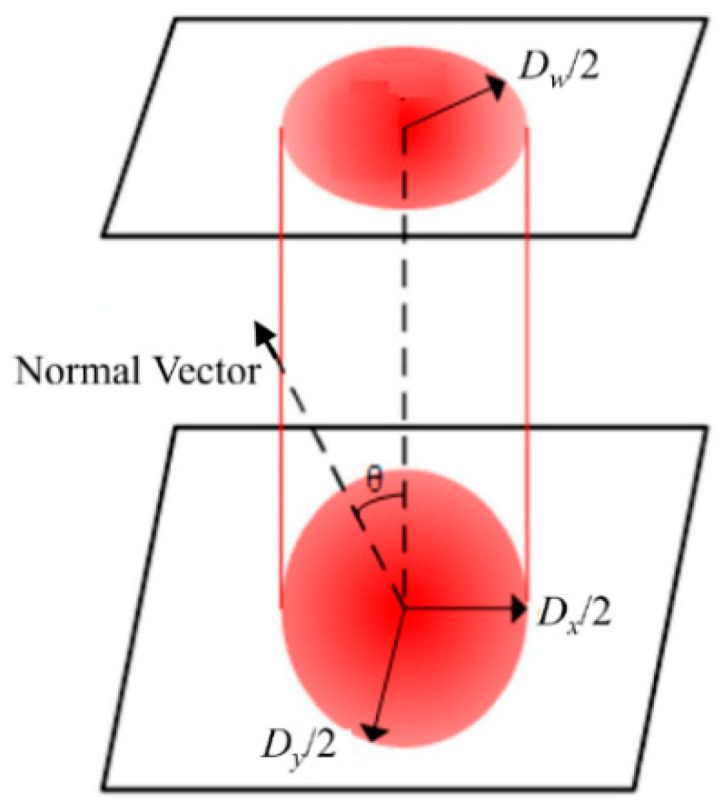
The projected spot size changing with the incident angle. *θ* is the angle between the laser light and the normal vector of the sample surface.

**Figure 6:**
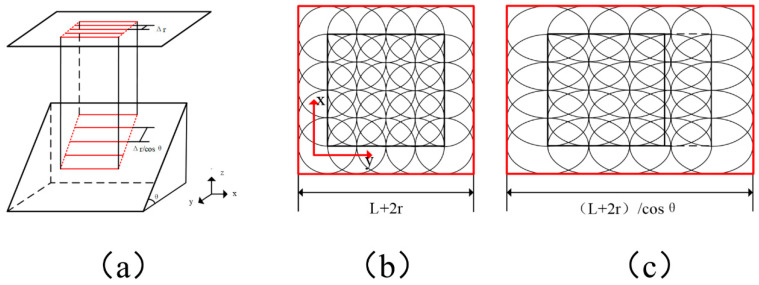
A schematic of the projected etching area changing with the incident angle (**a**), the ideal etching area (**b**) and the real elongated etching area (**c**). Due to distortion in the *y* direction, the etching accuracy was reduced.

**Figure 7:**
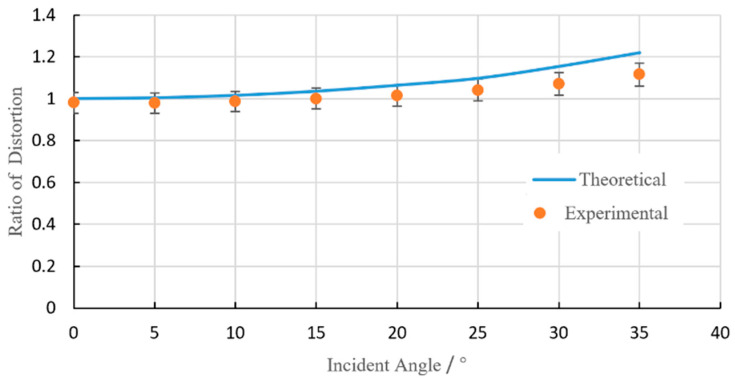
A comparison of etching distortion between the theory and the experiment.

When changing the incident angle, at least four issues may be changed: laser light polarization, laser fluence, reflectivity and the hatch space between the lines. As a result, the etching quality and accuracy are affected more or less. In this study:

(1) The polarization of the outcome laser beam was circular; thus, it should be irrelevant to the etching direction and result.
(2) When the laser light was normal to the surface (i.e., 0° of the incident angle), the projected area showed a circle shape. With the incident angle increasing, the projected area became elliptical, thus reducing the real laser fluence to the sample surface, as shown in Figure 5. In the figure, *d_w_* is the raw focused diameter, *θ* is the incident angle and *d_x_* and *d_y_* are the projected oval axis diameters. The reduction of laser fluence may have caused less etching depth. However, this variation was in the range of depth of focus, and the laser fluence was about six times that of the threshold fluence. For this situation, the fluence change due to the incident angle changing could be a minor factor.
(3) The reflectivity of the surface may have changed, which could also have had some effect on the etching [29,30]. For example, Chang et al. argued that the reflectivity was kept consistent and had not much difference on the ablation phenomenon when the incident angle was below 20° [31]. Their conclusion fitted this study very well. With the incident angle going up further, it seemed there was an obvious jump of the reflectivity, thus causing uneven etching results. A very similar phenomenon was reported by Liao et al. when studying laser welding [30]. All the experimental results suggested there should be quite little reflectivity change when the incident angle breaks through a threshold value, and this change caused a worse processing quality.
(4) When doing area etching, line-by-line hatching was usually adopted. With the incident angle varying, the hatch space was also affected (see Figure 6a for details). To simplify the situation, only the hatching distance in the *y* direction was elongated. After the projection transition, distortion happened: the square area changed to a rectangle. Consequently, the etching dimension in the *y* direction could have been affected from *L* + 2*r* to (*L* + 2*r*)/cos*θ* (see Figure 6b,c). In Figure 7, the calculated and measured dimension changes in the *y* direction are plotted, correspondingly. It can be seen that the measured experimental dimensions are smaller than the calculated ones. The smaller the incident angle was, the less the distortion was. The best coincidence was found at the incident angle of 10°.

### 3.2. The Influence of Pulse Overlap and Focal Spot Size on Etching Uniformity and Base Materials

**Figure 8:**
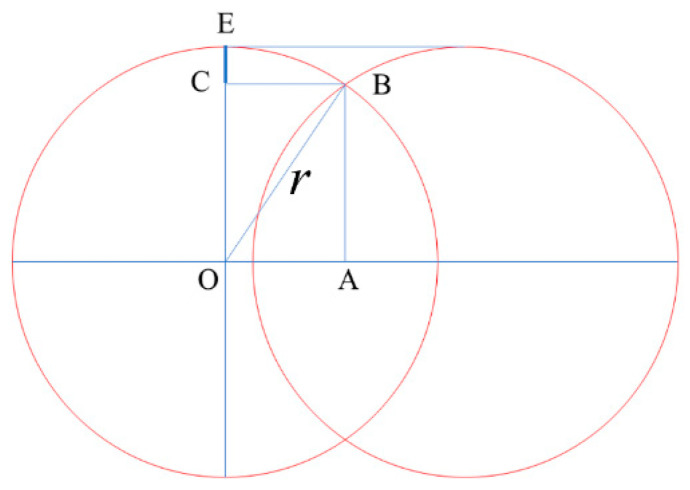
A schematic diagram showing the burrs dimension changing with the pulse overlap. As can be seen, the *EC* increases with the prolonging of *OA*.

Generally speaking, the electromagnetic absorption/filtration sensitivity of the FSSs relies largely on the etched area border uniformity rather than on the shape and dimensional accuracy. Pulse overlap and focal spot size are directly related to the etching area uniformity, which could be expressed by the burrs along the border edge. Figure 8 indicates the burrs variation with the pulse overlap changing. In the figure, *EC* presents the burrs dimension and could be calculated with the following equation:

(3) $$EC=r(1-\sqrt{1-(1-\varepsilon {)}^{2}})$$

where *r* is the radius of the spot size and *ε* is the percentage of pulse overlap, respectively. When *OA* = 0, two pulses totally overlapped and *ε* = 100%; when *OA* = *r*, two pulses just departed and *ε* = 0.

**Figure 9:**
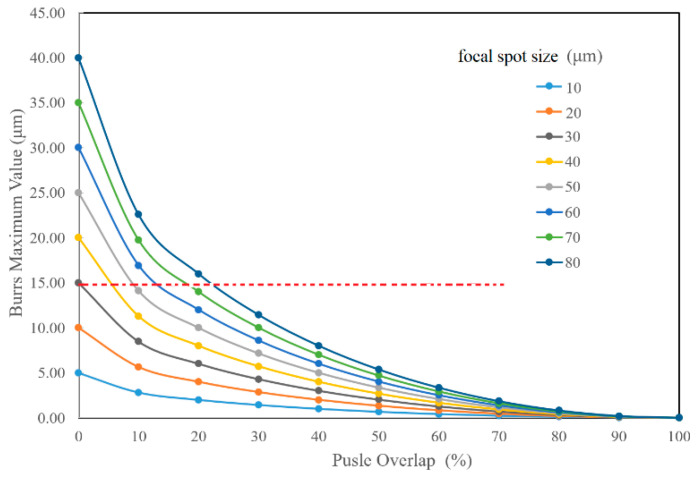
The relationship of the etching border burrs variation with the pulse overlap for different spot sizes. Under the current condition, an accuracy tolerance of 15 μm is required, which is shown with a red dashed line.

**Figure 10:**
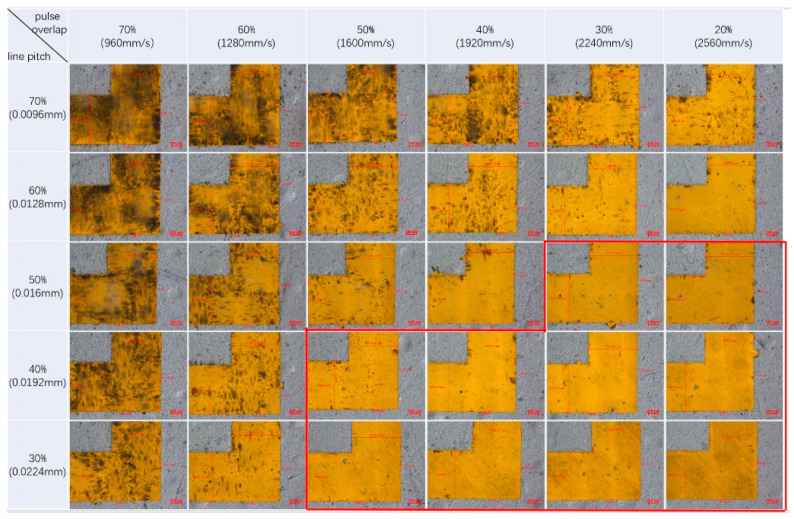
The etching results with different pulse overlaps by using a 32-μm focal spot size. The areas looped with red lines showed better etching results.

**Figure 11:**
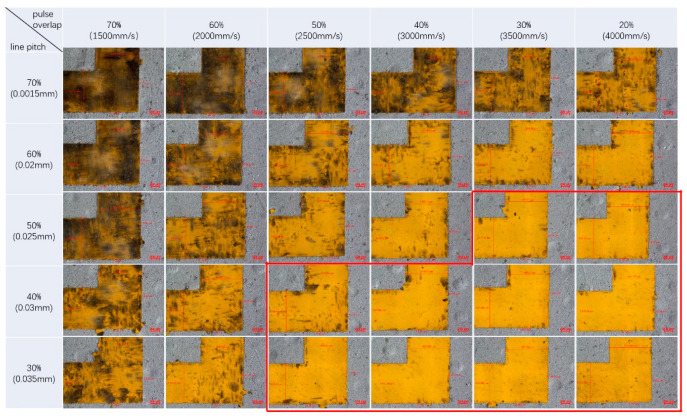
The etching results with different pulse overlaps by using a 50-μm focal spot size. The areas looped with red lines showed better etching results.

In Figure 9, the burrs fluctuation with pulse overlap for different spot sizes is shown. It is clear that the smaller the spot size and the more the pulse overlaps, the better the border uniformity. Thus far, a 15-μm border edge accuracy is an acceptable accuracy standard for FSS fabrication. It means the laser parameters below the red dashed line are favorable in Figure 9, theoretically. In order to further investigate the influence of the pulse overlap and focal spot size on the etching area uniformity, FSS pattern etching under spot sizes of 32 and 50 μm was carried out with the laser parameters in Table 2, and the results are given in Figure 10 and Figure 11. For the purposes of comparison, the laser fluences used in the experiments were almost the same as 20.64 J/cm^2^ for the 32-μm laser spot and 20.27 J/cm^2^ for the 50-μm laser spot. In the figures, the thermal effect is more and more obvious when the pulse overlap increased. The dark area indicates that some carbonized damage occurred. Additionally, the border burrs effects showed that the smaller spot size favored stable etching uniformity and stability. Optimal etching results usually occurred with pulse overlap at about ±40%, which has been explained in [32]. Combining the theoretical and experimental results, it could be inferred that the actual processing windows are smaller than those calculated, when the thermal effect and etching uniformity are considered, which are very important for FSS machining.

**Figure 12:**
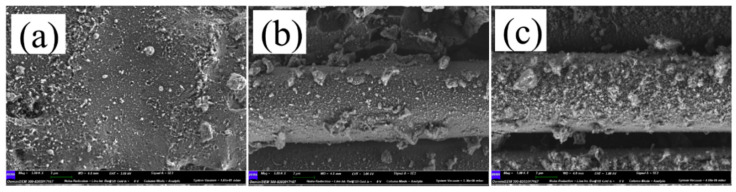
SEM images of the studied samples: (**a**) image of the unprocessed Al plate layer showing a relatively flat surface, (**b**) sample processed with a 32-μm focal spot size and (**c**) sample processed with a 50-μm focal spot size, showing silica fibers undamaged.

In order to further characterize the possible damage to base materials after laser etching, SEM pictures were captured and analyzed. In Figure 12a, the plain surface of the Al layer before etching is given. It looks very smooth, indicating good original flatness. According to observations of Figure 10 and Figure 11, samples etched with 30% pulse overlap and 40% line pitch showed good processing appearance and were then selected for SEM characterization. In Figure 12b,c, SEM images of base materials after laser etching were exposed, corresponding to the focal spot sizes of 32 and 50 μm, respectively. From the two figures, complete silica fibers still existed there because of the high melting point, revealing no obvious damage to the composite materials. Some resin residue on the fiber surface of the latter may indicate the etching consistence is inversely proportional to the focal spot size. On one hand, the smaller the focal spot size is, the better the etching consistence is; on the other hand, the smaller the focal spot size is, the lower the etching efficiency is. Therefore, a balance between the two sides should be considered.

## 4. Conclusions

Nanosecond laser etching of aluminum-plated composite materials applied to FSSs was demonstrated in this paper. The influence of the laser incident angle, pulse overlap and focal spot size were examined and discussed. The results led to the following conclusions:

- The laser incident angle plays a pivotal role to guarantee FSS etching quality and accuracy. It is found that an incident angle range of ±15° is suitable for 20-μm aluminum-layer composite material etching within the depth of focus.
- As the laser incident angle changes, the variation of laser light reflectivity and hatch space between lines causes the etching quality instability.
- Laser pulse overlap and focal spot size affects not only etching border accuracy and uniformity but also the material removal consistence. Combining the theoretical and experimental results, it is inferred that the actual processing windows are smaller than of those calculated, when thermal effect and etching uniformity are considered.
- In order to ensure etching area uniformity and no damage to base materials, a pulse overlap of 30~50% and a relatively small focal spot size are recommended from the experimental results.
- By reasonably selecting a processing window, optimal etching results on FSSs with the nanosecond fiber laser could be obtained. The method and experimental techniques in this study could be generalized to different FSS material composition processing.

## Figures and Tables

**Table 1:** Laser parameters for the study of the laser light incident angle’s influence.

Spot Size (μm)	Pulse Width (ns)	Repetition Rate (kHz)	Scanning Speed (mm/s)	Peak Power (Mw)	Peak Fluence (J/cm^2^)
32	100	100	1920	16.6	20.64

**Table 2:** Laser parameters for the study of the focal spot size and the pulse overlap’s influence.

Spot Size (μm)	Pulse Width (ns)	Repetition Rate (kHz)	Scanning Speed (mm/s)	Peak Power (Mw)	Peak Fluence (J/cm^2^)
32	100	100	960–2560	16.6	20.64
50	100	100	1000–4000	39.8	20.27
